# Triple-negative breast cancer: A run-through of features, classification and current therapies

**DOI:** 10.3892/ol.2021.12773

**Published:** 2021-05-05

**Authors:** Meghana Manjunath, Bibha Choudhary

**Affiliations:** 1 Department of Biotechnology, Institute of Bioinformatics and Applied Biotechnology, Bengaluru, Karnataka 560100, India; 2 Manipal Academy of Higher Education, Manipal, Karnataka 576104, India

**Keywords:** triple-negative breast cancer, targeted therapy, metastatic, overall response rate, clinical trials

## Abstract

Breast cancer is the most prevalent cancer in women worldwide. Triple-negative breast cancer (TNBC) is characterized by the lack of expression of estrogen receptor, progesterone receptor, and human epidermal growth factor receptor 2. It is the most aggressive subtype of breast cancer and accounts for 12–20% of all breast cancer cases. TNBC is associated with younger age of onset, greater metastatic potential, higher incidence of relapse, and lower overall survival rates. Based on molecular phenotype, TNBC has been classified into six subtypes (BL1, BL2, M, MES, LAR, and IM). TNBC treatment is challenging due to its heterogeneity, highly invasive nature, and relatively poor therapeutics response. Chemotherapy and radiotherapy are conventional strategies for the treatment of TNBC. Recent research in TNBC and mechanistic understanding of disease pathogenesis using cutting-edge technologies has led to the unfolding of new lines of therapies that have been incorporated into clinical practice. Poly (ADP-ribose) polymerase and immune checkpoint inhibitors have made their way to the current TNBC treatment paradigm. This review focuses on the classification, features, and treatment progress in TNBC. Histological subtypes connected to recurrence, molecular classification of TNBC, targeted therapy for early and advanced TNBC, and advances in non-coding RNA in therapy are the key highlights in this review.

## 1. Introduction

Breast cancer is a heterogeneous disease with varying biological and clinical characteristics. It is the most common cancer among women worldwide, accounting for 25% of all cancer cases (1). According to GLOBOCAN 2020, the incidence and mortality of breast cancer reported worldwide were 34,65,951 new cases and 11,21,413 deaths, respectively; in India, 1,204,532 new cases and 436,417 deaths were recorded in 2020 (2).

Immunohistochemical analysis of breast tumors is the gold-standard method used in clinics to classify them based on the hormone receptor expression for improved therapeutic decisions. Based on this, breast cancer can be broadly grouped into five types, namely: i) Progesterone receptor (PR)-positive, estrogen receptor (ER)-positive and human epidermal growth factor 2 (Her2)-negative (luminal A); ii) ER-positive, PR-positive/negative and Her2-positive (luminal B); iii) Her2-overexpressing, ER- and PR-negative; iv) ER-, PR- and Her2-negative (basal-like or triple-negative), and v) normal-like (expression status similar to luminal A and resemble normal breast profile) (3–5). Additionally, molecular breast cancer analysis identified a distinctive phenotype with low claudin expression, immune receptor, and EMT markers expression (6). Cancer types with the claudin^low^ phenotype are highly metastatic and associated with poor prognosis (7). Her2-overexpressing cancer also displays high metastasis and poor prognosis (8). Among ER-positive subtypes, luminal B is associated with a significantly worse prognosis than luminal A (9,10). Patients with basal subtypes of cancer with BRCA1 mutations have a poor prognosis (9).

Based on specific gene expression patterns, breast cancers are categorized into five intrinsic or molecular subtypes. Among the intrinsic subtypes, basal-like triple-negative breast cancer (TNBC) accounts for 12–20% of breast cancers (11). TNBC has drawn specific attention due to the lack of expression of all three receptors (ER, PR, and Her2). Thus, it cannot be treated using anti-estrogen hormonal therapies or trastuzumab (12). Morphologically, TNBC is characterized by hyperdense masses without calcification, usually occurring in women <50 years of age. Histological features include significant lymphocyte infiltration, central necrosis, pushing tumor borders, and fibrosis (13). Cytokeratins, fascin, epidermal growth factor receptor (EGFR), caveolin, and vimentin are usually expressed in basal-like TNBC (14,15). TNBC is challenging to treat, as it is quite complex due to poor cell differentiation, molecular heterogeneity, and rapid metastasis, often leading to chemoresistance and recurrence of the disease (16). Fast relapse and invasions are common features of TNBC tumors and show poor prognosis (17). Recent advances in omics technologies have provided insight into the molecular mechanisms underlying TNBC (18). The present review focuses on the different subtypes of TNBC and therapeutic approaches currently employed in the treatment of TNBC.

## 2. Histology-guided classification of TNBC

Histologically, most TNBC is categorized as no special type (IDC-NST) (17). Most IDC is characterized by pleomorphic cells with prominent nucleoli. The cells are organized into diffuse sheets, cords, nests with ductal differentiation. The rest of the tumors are categorized into 47 specific subtypes, such as invasive lobular carcinoma (relatively common), metaplastic carcinoma, medullary carcinoma, mucinous carcinoma, adenoid cystic carcinoma, secretory carcinoma, acinic cell carcinoma, neuroendocrine tumors, as well as the rarest glycogen-rich clear cell carcinoma (19,20).

**Figure 1.**
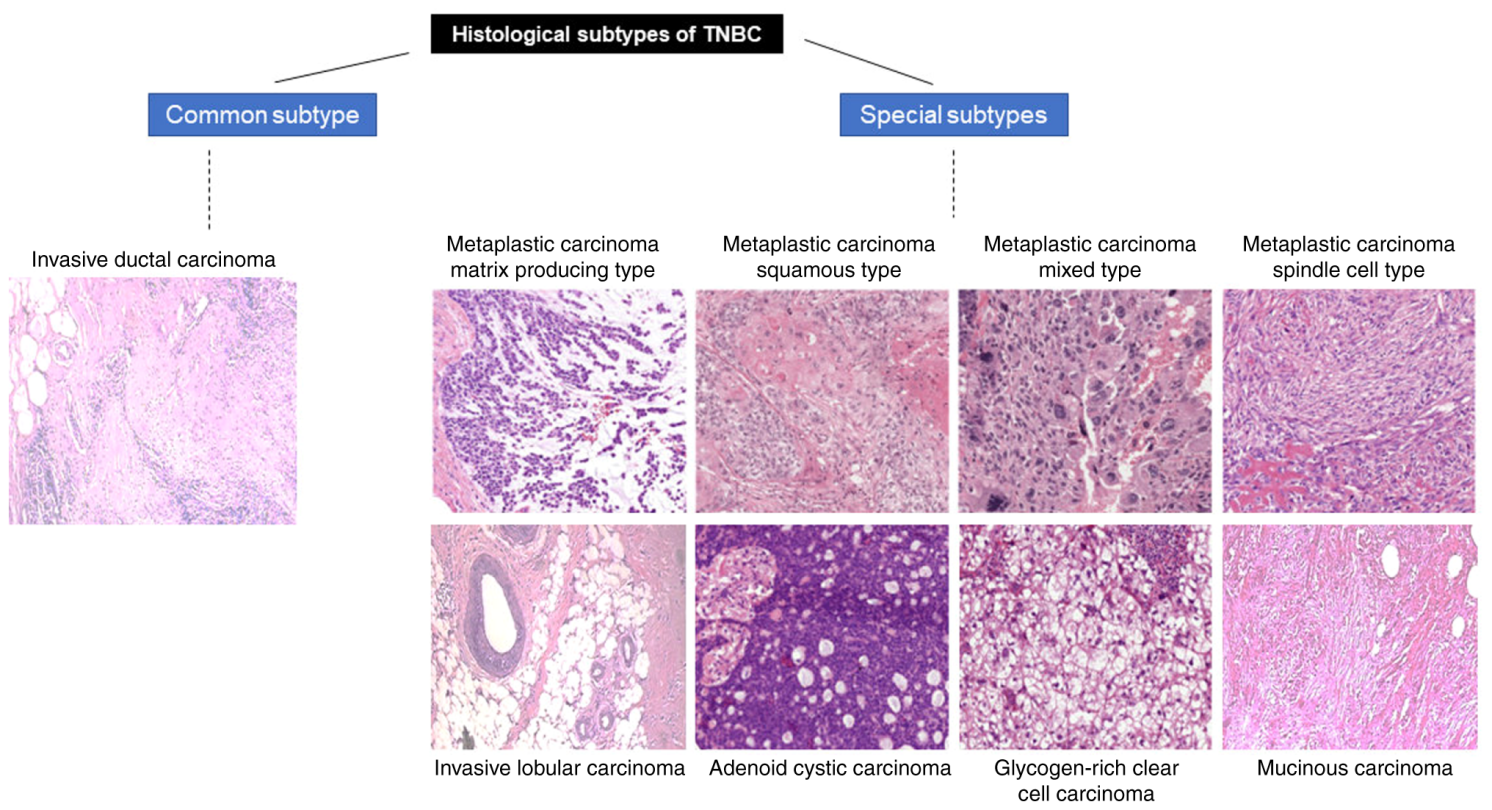
Histological classification of TNBC. Among the histological subtypes, invasive ductal carcinoma is the most common. Metaplastic, adenoid cystic, invasive lobular, mucinous and glycogen-rich clear cell carcinoma are rare subtypes of carcinoma. Metaplastic carcinoma can be further divided into matrix-producing, squamous, mixed and spindle-cell type, depending on the cell type (15). The permission to use this figure is licensed under a Creative Commons Attribution-ShareAlike 4.0 International License with Elsevier. TNBC, triple-negative breast cancer.

Among these specific subtypes (Fig. 1), medullary breast carcinoma occurs in <1% of patients and shows distinctive features, such as high lymphoplasmacytic infiltration, overexpression of BCLG (a pro-apoptotic gene); it bears more losses of heterozygosity than other subtypes and is immunomodulatory (21,22). It is associated with better outcomes compared with other TNBC subtypes (22). Metaplastic carcinoma presents unique pathologic features, where the glandular component may be partially or completely replaced by a non-glandular component(s), and based on their differentiation status further divided into i) Squamous type, tumor with keratinization and squamous differentiation; ii) matrix-producing type, tumor with more cells in the periphery; iii) mixed type, tumor showing both squamous differentiation and large high-grade cells with pleomorphic nuclei; and iv) spindle-cell type, tumor with storiform-like spindle cells. These metaplastic tumors harbor mutations in the PIK3CA, Wnt (Wingless-Type MMTV Integration Site Family) signaling pathway genes and display a unique copy number alteration pattern (23–25). Adenoid cystic carcinoma (ACC) is characterized by the presence of dividing epithelial cells and myoepithelial cells producing mucinous membrane. ACC occurs in 0.1% of patients with basal-like features (26,27) and expresses markers, such as cytokeratin 5, −5/6, −14 and −17 (28). Secretory carcinoma is characterized by microcystic, solid and tubular architecture and presence of vacuolated tumor cells producing intracellular and extracellular secretions. It occurs in <1% of the patients and is referred to as juvenile carcinoma, as it is common in adolescents and often reported to have favorable outcomes. It is also characterized by ETV6-NTRK3 fusion (29–31). The rarest among all the subtypes is glycogen-rich clear cell carcinoma, in which the tumors appear in sheets and cells are polygonal in shape, with a clear cytoplasm and the presence of glycogen (32). In these sheets, there are areas of lymphocytic infiltration and plasma cells.

Among the histological subtypes, adenoid cystic carcinoma has a median recurrence of only 2 months, and metaplastic carcinoma has ~9.9 months (33), compared to IDC-NST and matrix-producing metaplastic carcinoma, which are less aggressive, with 34 and 31.4 months of median time to recurrence, respectively (20).

Although the histological assessments were pointing to the presence of WBCs in and around the TNBC subtypes, focus on the presence of WBCs has led to the identification of TILS and TAMs, which are the parameters defining prognosis and therapy of TNBC. The TNBCs might be immunogenic due to mutations that lead to aberrant protein expression on the cell membrane (34). Tumor-infiltrating lymphocytes (TILs) are white blood cells that migrate towards the tumor from the bloodstream via the newly formed blood vessels (angiogenesis), which cancer cells use for their nutritional and oxygen requirements (35). They consist of a mixture of B cells, macrophages, natural killer cells and are dominated by T cells (35). TILs are present in ~20% of TNBC tumors and carry a pivotal prognostic and predictive value (36). The presence of TILs indicates a good prognosis (37). High number of TILs indicate that there is an equilibrium between the immune status and cancer (38). The ratio of cancer cells: TILs is tilted towards TILs after surgical removal of a tumor, resulting in an improved prognosis in TNBC (38). A high mutation load and clonal heterogeneity are associated with a low number of TILs, which may provide an escape route to tumor cells from immune surveillance (39). However, in addition to TILs, the tumor microenvironment components also influence the outcome of patients with TNBC (39). Relapsing patients with TNBC have been shown to have low levels of TILs and a high number of CD163^+^ tumor-associated macrophages (TAMs) compared with that of patients without relapse (39). High levels of CD8^+^ T cells may reflect improved sensitivity to chemotherapy, whereas high levels of TAMs correlate with poor patient outcomes (36). Nevertheless, a previous study in TNBC has reported paradoxical findings, with high levels of CD8^+^ T cells in the tumor stroma leading to the low infiltration of the tumor epithelium, thereby indicating a poor outcome (40). Therefore, immunohistological assessment for TILS or TAMS will help develop immunotherapies detailed in section 7.

## 3. Multiomics-guided molecular classification of TNBC

Profiling based on gene expression has led to improved insight into tumor heterogeneity at the molecular level and has generated an impartial classification (Fig. 1). The PAM50 microarray set of 50 genes is used to identify breast cancer intrinsic subtypes (41). A set of 374 TNBC samples taken from 14 microarray datasets was analyzed to characterize TNBC subtypes using PAM50. The results from this analysis categorized most of the TNBC as basal-like (80.6%). The rest of the tumours were classified as Her2-positive(0.2%), normal-like (14.6%), luminal B (3.5%) and luminal A (1.1%) (Table I) (41).

Lehmann *et al* (42) performed gene expression profiling of 2,188 genes from 587 patients with TNBC and classified TNBC into six new groups, namely, basal-like 1 (BL1), basal-like 2 (BL2), immunomodulatory (IM), luminal androgen receptor (LAR), mesenchymal stem cell-like (MSL) and mesenchymal (M). The rest was classified as an unstable type (UNS/UNC). Each subtype had its characteristic feature. Basal-like was the most common type of TNBC (BL1, 22%; BL2, 12%) and was characterized by high Ki67 and DNA damage response levels. The IM subtype (18%) had basal-like characteristics with activation of IFNα and IFNγ signaling and high cytotoxic T-lymphocyte associated protein 4 gene expression. Mesenchymal subtypes (M, 21%; MSL, 10%), along with cell differentiation pathways, showed deregulation of EGFR, calcium signaling, MAPK, and PI3K signaling. In the LAR subtype (9%), an ~10-fold increase in androgen receptor (AR) expression was seen, compared with other subtypes. Activation of various pathways, such as steroid synthesis and FOXA1 and ERBB signaling, were observed in this subtype (Table I) (42,43).

Burstein *et al* (43) used a non-negative matrix factorization method to derive a panel consisting of 80 core genes that divided TNBC into four subtypes, luminal-AR (LAR), mesenchymal (MES), basal-like immune-suppressed (BLIS), and basal-like immune-activated (BLIA). BLIA has the best disease-free survival outcome compared to other subtypes (44). Based on DNA copy number, these subtypes can be placed into two groups, LAR or others (Table I) (31).

Liu *et al* (45) performed mRNA and long non-coding RNA (lncRNA) expression analysis in 165 TNBC tumor samples at Fudan University Shanghai Cancer Centre. The tumor samples were categorized into four subtypes (IM, LAR, MES, and BLIS subtypes), consistent with the classification by Burstein *et al* (43). The IM subtype comprised of genes related to immune functions such as CCR2, CXCL13, CXCL11, CD1C, CXCL10, and CCL5, along with ENST00000443397 long ncRNA. In contrast, the LAR subtype had enrichment of hormone regulation signaling and ENST00000447908 lncRNA (45). The MES subtype expressed lncRNA NR_003221 together with genes and pathways that promoted epithelial-to-mesenchymal (EMT) transition. Pathways and molecules such as DNA repair, replication, and mitosis, lncRNA TCONS_00000027 were enriched in the BLIS subtype (45,46).

Genomic/transcriptomic data from a set of 997 primary tumors were extracted, and an integrated analysis was performed by Curtis *et al* (47). A set of 995 tumors from the Molecular Taxonomy of Breast Cancer International Consortium (METABRIC) cohort was used as a validation set that divided TNBC into ten groups, named Integrated Clusters (IntClust) 1–10 (47). Basal-like breast cancer mostly fell in IntClust 4 and 10 (~80%). IntClust 4 is known to have greater TIL counts, while IntClust 10 subtype can display genomic instability and chromosomal aberrations (Table I) (47–49).

## 4. Molecular aberrations in TNBC

Through whole-exome and whole-genome data, it is evident that most of the genetic alterations in TNBC are copy number alterations and somatic mutations (40). The BRCA1 and BRCA2 tumor suppressor genes are required for the maintenance of genomic stability. These genes play a role in DNA repair and replication error control (50,51). A total of 10% of patients with TNBC are known to harbor germline mutations in BRCA1 or BRCA2 (12,26,27). The lifetime risk of breast cancer becomes 60–70% in the presence of such mutations (52). Gene alterations leading to homologous recombination (HR) defects other than germline BRCA mutations are termed ‘BRCAness’ (53). Moreover, ~35% of TNBC tumors show abnormalities in the HR pathway, making them sensitive to poly (ADP-ribose) polymerase (PARP) inhibitors and DNA-damaging agents (54).

**Figure 2.**
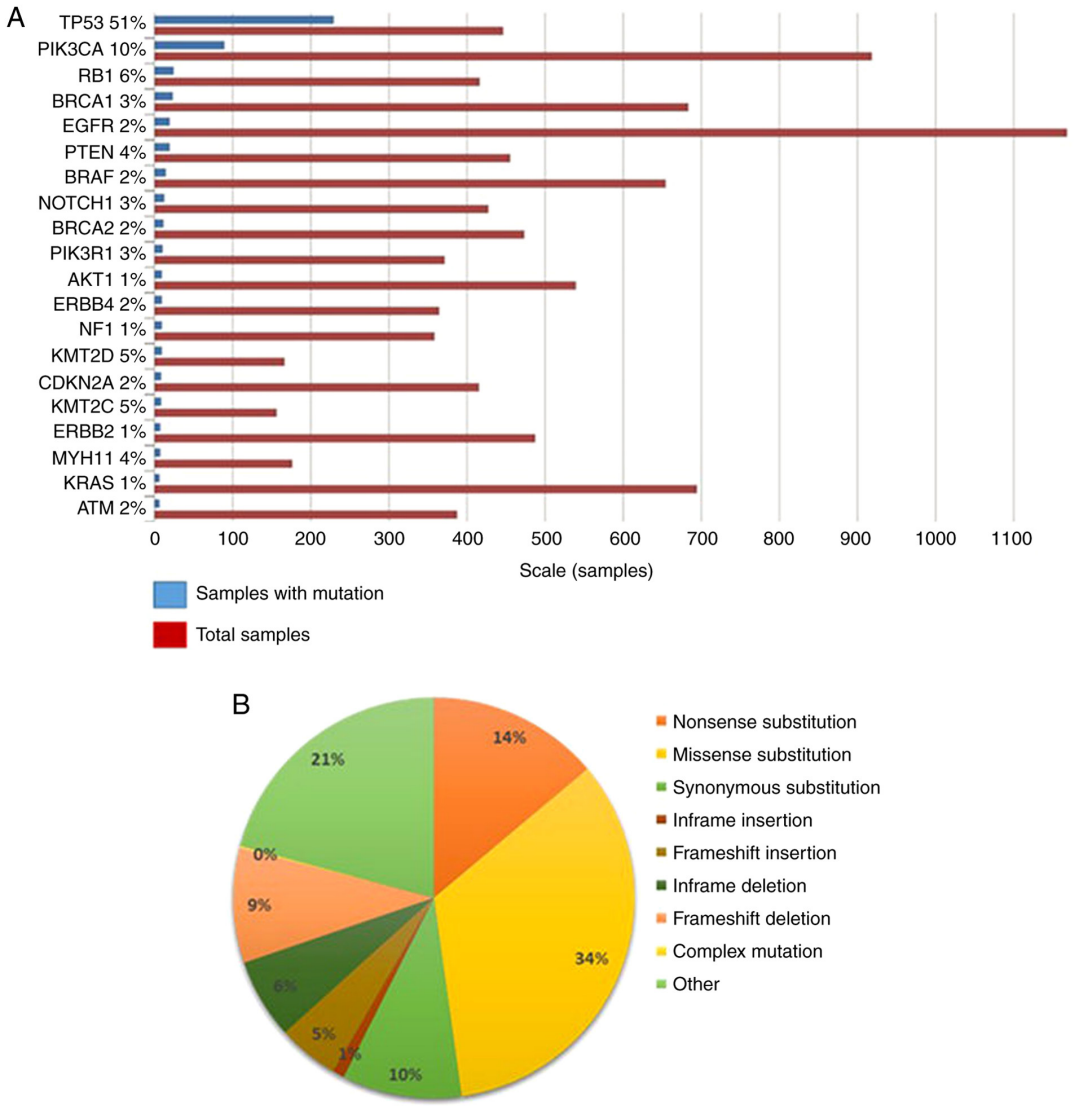
Mutation status of the top 20 genes in triple-negative breast cancer identified in the Catalogue Of Somatic Mutations In Cancer. (A) Bar graph showing the number of samples harbouring mutations in the top 20 genes with most mutations. The blue bar indicates samples with mutations, whereas the red bar indicates the total number of samples. The percentage of occurrence of mutation is also shown. TP53, PI3K, BRCA1 are among the top genes in the panel with a high number of mutations. (B) Pie chart classifying the type of mutations observed in all genes. Most of the mutations fall under nonsense and synonymous type. Other mutation types, such as frameshift and in-frame mutations, were also observed.

Other common mutations observed in TNBC patients include those in TP53 (50–60%) and PIK3CA (~10%) (18,42). An analysis from the Catalogue of Somatic Mutations in Cancer (COSMIC) database revealed that the top genes mutated in TNBC, apart from BRCA1/2, TP53, and PIK3CA, were RB1, PTEN, NOTCH1 and BRAF (Fig. 2A). Among the point mutations observed, 34% of them were nonsense substitutions (where a base change leads to a stop codon in the DNA sequence), 21% were synonymous mutations (where a change in a base in the exon of a coding gene does not change the structure of the protein) (Fig. 2B). The rest of the mutations were missense mutations, frameshift insertion/deletions, and in-frame insertions/deletions. In the metastatic disease setting, genes from HR repair showed a larger frequency of biallelic loss-of-function mutations than in early TNBC (55).

Integrated analysis of The Cancer Genome Atlas (56) has demonstrated deletions in PTEN, DUSP4, and INPP48 involved in the PI3K-AKT pathway. Gene amplifications were seen in MYC, PIK3CA, KRAS, BRAF, FGFR, MET, and EGFR. Mutations in genes, such as ERBB2, AKT1, ATR, MAP3K1, CDKN2A, ATM, and NOTCH2 (18,42,51), were also observed. Based on the mutation signatures obtained from whole-genome sequencing of 560 tumors, TNBC could be classified into four mutation subtypes, namely, APOBEC-based signatures, HR deficiency-based signature (signature 3), Clock-like signatures (signatures 1 and 5), and mixed (no prominent signature) (56,57). These mutations suggest that DNA repair, the PI3K/AKT pathway, cell cycle checkpoints, and Notch signaling are possible druggable pathways in TNBC (58).

## 5. Circulating tumor cells (CTCs) in TNBC

Recently, much focus has been put on bringing liquid biopsies, such as circulating tumor cells (CTC) and circulating tumor DNA (ctDNA), into the clinical setting for diagnostic and prognostic use (59). CTCs are nucleated cancer cells present in the bloodstream that can be detected using techniques, such as reverse transcription-quantitative PCR, flow cytometry, and immunohistochemistry (60). Tumor cells that undergo necrosis or apoptosis release DNA fragments into the plasma are referred to as ctDNA (61). In breast cancer, ctDNA and CTCs have been studied as potential biomarkers for prognosis (60). Stover *et al* (62) performed studies in metastatic breast cancer patients receiving chemotherapy and identified an association between CTCs and ctDNA and tumor burden, indicating that these could be used to measure early-treatment response in patients. A retrospective study in 164 patients with metastatic TNBC revealed that >10% of patients with ctDNA had worse disease-free survival (62). A study by Bidard *et al* (63), with metastatic breast cancer, revealed that patients with CTC levels >5 per 7.5 ml were associated with lower progression-free survival (PFS) and OS compared with patients who had CTC levels <5 per 7.5 ml. Cristofanilli *et al* (64) reported that CTC counts could be utilized to classify metastatic patients into two groups. Patients with CTCs levels >5 per 7.5 ml were categorized as aggressive stage IV and those <5 per 7.5 ml as indolent stage IV (64). ctDNA has been associated with chemotherapy in studies by Riva *et al* (65), in which ctDNA-positive patients before and after chemotherapy experienced poor OS and disease-free survival (DFS). Additionally, Radovich *et al* (66) reported that patients with early-stage TNBC and positive ctDNA after chemotherapy had a higher risk of disease relapse. Therefore, liquid biopsies are being developed as a non-invasive method to study recurrence, treatment response, and survival in the clinical setting.

## 6. Conventional mode of treatment in TNBC

**Figure 3.**
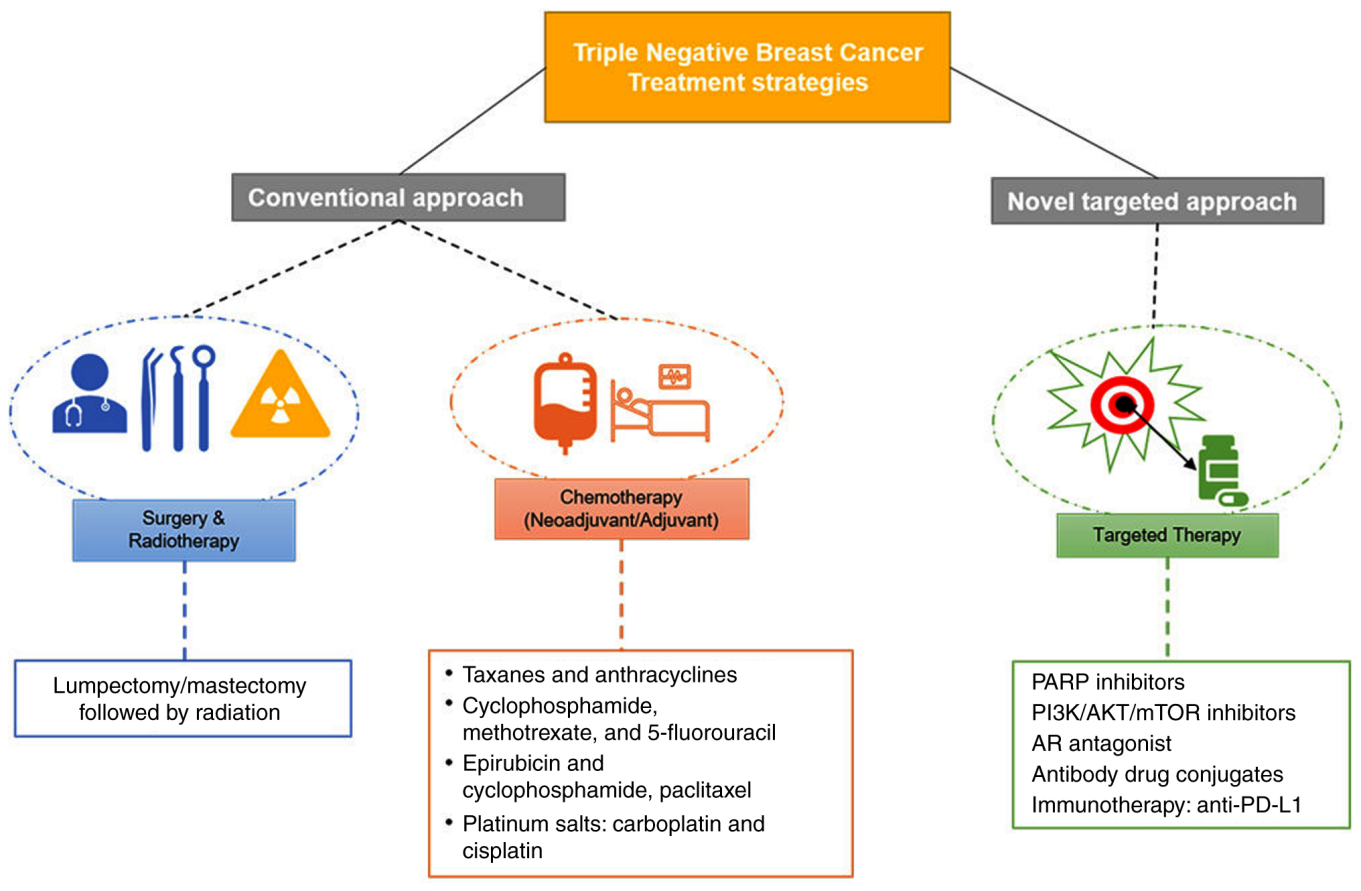
Different modes of treatment employed in TNBC therapy. The traditional method of treating cancer (surgery and radiotherapy) is still the primary mode of initial treatment followed by chemotherapy. Taxanes and anthracyclines are common chemotherapeutic agents used for the treatment of TNBC along with platinum salts. Recently, due to the development of omics technology, targeted therapy has become a novel way of treating cancer. AR, androgen receptor; PARP, poly (ADP-ribose) polymerase; PD-L1, programmed death ligand 1; TNBC, triple-negative breast cancer.

TNBC treatment involves a combination of surgery, radiotherapy, and chemotherapy. New methods, such as targeted therapy and immunotherapy, have been developed to improve patient survival and prognosis. Lumpectomy and mastectomy are the surgical procedures performed for TNBC patients and are usually followed by radiotherapy and chemotherapy (67). Neoadjuvant therapy is given before the surgery, which may help shrink the tumor size and avoid mastectomy (Fig. 3) (61). Taxanes and anthracyclines form the current standard of care for TNBC in both the neoadjuvant and the adjuvant settings. Epirubicin and doxorubicin are the most common anthracyclines (anticancer antibiotics known to disrupt DNA replication and mitochondrial functions to activate apoptosis) (68,69). Taxanes block angiogenesis by inhibiting epidermal growth factor receptor signaling (70).

Paclitaxel and docetaxel are familiar examples of taxanes used in the first line of therapy (71). TNBC shows a 40% pathological complete response (pCR) for taxane and anthracycline-based therapy in the neoadjuvant setting (72–74). Adjuvant therapy guidelines are usually identical for all the subtypes of breast cancer and TNBC. Chemotherapy in the adjuvant setting is recommended for tumors >0.5 cm in size, as they exhibit increased aggressiveness, with a faster growth rate and metastasis (75). Anthracycline chemotherapy (cyclophosphamide and 5-fluorouracil) in patients with metastatic TNBC exhibited a response to survival within 22 months (69). However, acute toxicity is a major concern with anthracycline-based chemotherapy (76). Metastatic patients who develop resistance to anthracycline have shown sensitivity to capecitabine, gemcitabine and vinorelbine (77–79). The combination of docetaxel with capecitabine has improved the OS of patients with metastatic TNBC (78).

Carboplatin and cisplatin are platinum salts that are used in the treatment of TNBC. These generate DNA lesions, and apoptosis occurs in cells unable to repair these breaks (80). For TNBC, carboplatin as a neoadjuvant addition increases the response rate from 37 to 52.1% (81). A phase-II study of 86 patients evaluating the efficacy of platinum monotherapy demonstrated a 32% overall response rate (ORR) for cisplatin and 19% for carboplatin in early TNBC. Patients with BRCA1/2 mutations showed an improved response compared with patients without BRCA1/2 mutations (82). Moreover, phase-II trials showed an improved ORR of 72% in metastatic patients with BRCA mutation with neoadjuvant cisplatin monotherapy (83,84). Recently, the PEARLY trial (NCT02441933) has explored combination therapy of taxanes and carboplatin in the neoadjuvant setting (85). Carboplatin with docetaxel or paclitaxel combination has demonstrated promising efficacy in patients with TNBC and brain metastasis (86). Although TNBC is sensitive to chemotherapy, early relapse is a major concern (75). Therefore, optimizing a tailored standard regime to address chemotherapy issues, such as toxicity, and relapse has led to customizing personalized therapy based on tumor type.

## 7. Emerging role of targeted therapy as a strategy to treat TNBC

Therapies targeted to TNBC are being developed based on the expression of specific pathways and genes. Targeted therapy focuses on customizing cancer therapy to an individual patient's tumor (87,88). TNBC being heterogenous, targeting alterations specific to the tumor would be the most effective treatment option. A study using genomics and transcriptomics has led to identifying molecular markers that could be effectively targeted in TNBC (89). PARP inhibitors, PI3K/AKT inhibitors, and anti-androgen therapy are under clinical investigation (Fig. 3) (58).

### 

#### PARP inhibitors for patients with a BRCA1/2 mutation

**Figure 4.**
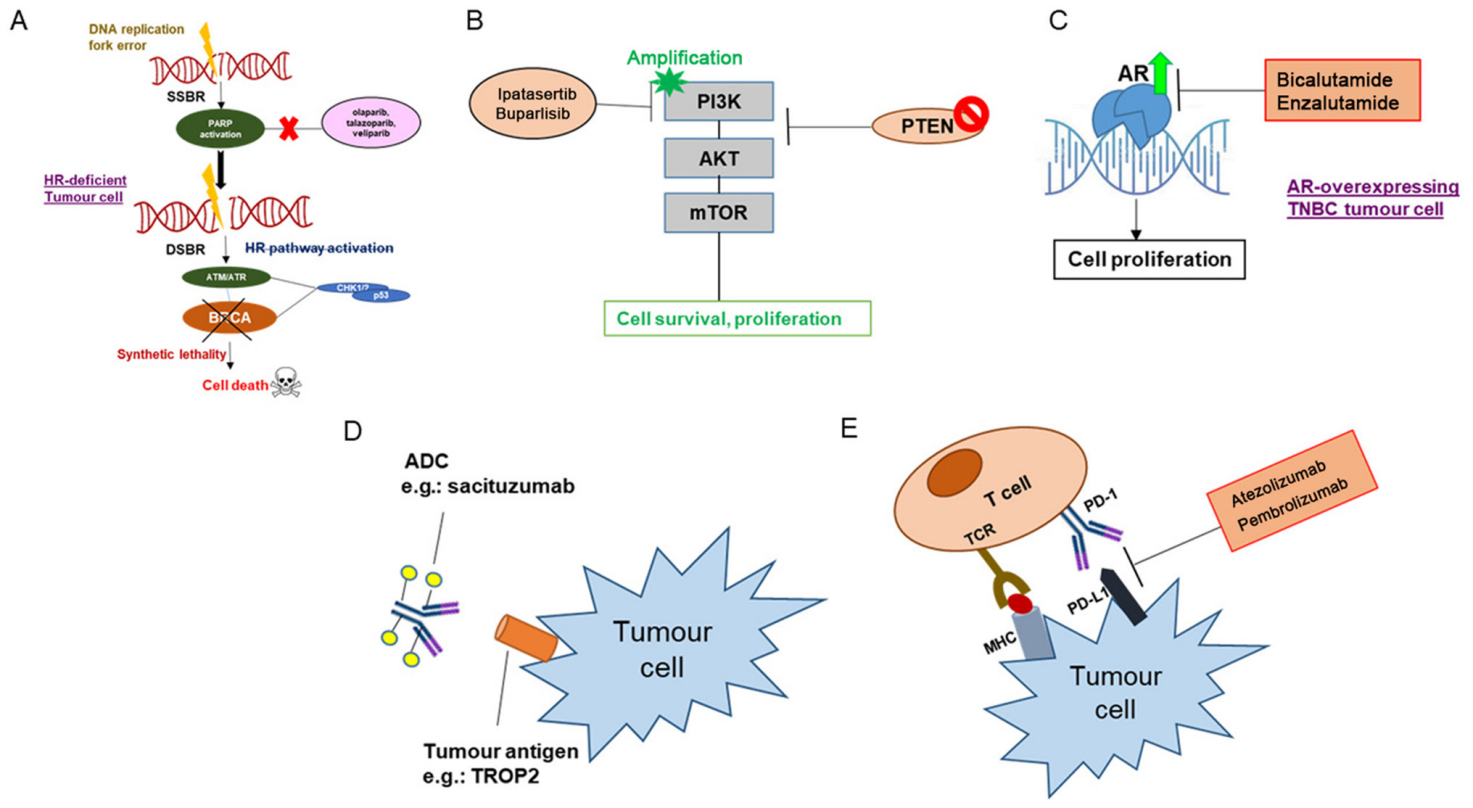
Targeted therapies currently explored for TNBC. (A) PARP inhibitors, such as olaparib and veliparib are currently under clinical trials to bring about synthetic lethality in homologous recombination-deficient TNBC harbouring BRCA1 mutations. (B) PI3K-activated TNBC with loss of PTEN can be treated with buparlisib or ipatasertib, which inhibit the PI3K enzyme. (C) In the LAR subtype of cancers, where the expression of AR is very high, AR blockers like bicalutamide and enzalutamide have made their way. (D) Antibody-drug conjugates are specific to cell-surface molecules and linked to a potent cytotoxic agent that kills the cancer cell. (E) PD-L1 blockade using atezolizumab has shown positive results in a clinical trial on patients with TNBC. AR, androgen receptor; DSBR, double-strand break; MHC, major histocompatibility complex; PARP, poly (ADP-ribose) polymerase; PD-1, programmed death-1; PD-L1, PD ligand 1; SSBR, single-strand break; TCR, T cell receptor; TNBC, triple-negative breast cancer; LAR, luminal androgen receptor; ATM, ataxia telangiectasia mutated; ATR, ataxia telangiectasia and rad3-related protein; HR, homologous recombination; ADC, antibody-drug conjugates; TROP-2, tumour associated calcium signal transducer 2.

PARP is expressed in ample amounts as a nuclear enzyme that plays a critical role in DNA repair, cell proliferation, and signaling. It transfers ADP-ribose to target proteins from NAD^+^ and ribosylates them (90). In response to DNA damage, PARP is known to activate the DNA repair process through poly (ADP)-ribosylation of multiple nuclear proteins that play a role in chromatin architecture and DNA metabolism (91). Therefore, PARP inhibition leads to the accumulation of double-strand breaks (DSBs) in cells undergoing replication. The presence of wild-type BRCA1/2 in cells results in a homologous recombination mode of repair of DSBs. However, in the cells deficient of BRCA1/2, homologous recombination is disrupted, and PARP repairs the breaks (92–94). Therefore, in these BRCA1/2-deficient cases, inhibiting PARP will result in severe, selective toxicity called ‘synthetic lethality’ (95). Using PARP inhibitors in treatment sensitizes the tumor cells to chemotherapy and radiotherapy, causing synthetic lethality in patients with hereditary BRCA1/2 mutations identified in several TNBC subtypes (Fig. 4A) (96).

Olaparib and talazoparib are two of the PARP inhibitors approved by the United States Food and Drug Administration (FDA) for use in patients with deficient BRCA1/2 in metastatic Her2-negative breast cancer as a single agent, based on the phase-III OlympiAD and EMBRACA clinical trials (86–88). Patients with a germline BRCA1/2 mutation (gBRCA1/2^+^) with metastatic breast cancer were grouped into 2:1 to olaparib vs. chemotherapy (capecitabine, eribulin, or vinorelbine) of physician's choice in OlympiAD trial (NCT02000622) (97,98). The ORR was 59.9% in the TNBC patient subgroup for olaparib (n=102) and 29.9% in the case of patients who underwent chemotherapy (n=48). Olaparib showed less toxicity in tumorgrade3 and 4 patients than the chemotherapy arm (98,99). In the EMBARCA trial (NCT01945775), gBRCA1/2^+^ metastatic patients were given 2:1 Talazoparib 1 mg daily vs. chemotherapy of physician's choice. The ORR was 62.6% in patients given with Talazoparib (n=219) and 27.2% in patients treated with chemotherapy (n=144) (99). Several other PARP inhibitors are currently under phase-II/III clinical trials, including veliparib (NCT02163694) and niraparib (NCT01905592) (100–103).

PARP inhibitors are being investigated in combination with chemotherapy and immunotherapy. BrighTNess trial (NCT02032277) is a phase-III trial for stage-II and -III TNBC evaluating the combination of carboplatin with the PARP inhibitor veliparib followed by doxorubicin (104). The ongoing phase-I/II trial (MEDIOLA trial) involves a combination of olaparib and anti-PDL1 checkpoint inhibitor durvalumab (105). The phase-III OlympiA trial (NCT02032823) for early TNBC is currently assessing patients with BRCA1/2 mutation treated with olaparib as monotherapy following neoadjuvant chemotherapy (106). PARTNER (NCT03150576) (107) is a phase-II/III trial that is currently ongoing checking the efficacy of olaparib and carboplatin combination in a neoadjuvant setting (107). Table II summarizes the clinical trials taken from clinicaltrials.gov.

#### PI3K/AKT inhibitors for PTEN low TNBC

PI3K/AKT pathway is involved in cell growth and glucose metabolism. Under normal conditions, growth factors, such as insulin-activated receptor tyrosine kinases (RTKs) result in PI3K activation (108). This is followed by phosphorylation of phosphatidylinositol-4,5-trisphosphate (PIP2) by PI3K and conversion to phosphatidylinositol-3,4,5-trisphosphate (PIP3) (109). AKT binds to membrane-bound PIP3, bringing AKT close to phosphoinositide-dependent kinase 1 (PDK1) (110). PDK1 phosphorylates AKT resulting in the activation of multiple downstream pathways like cell growth, cell cycle, and metabolic pathways. This pathway is negatively regulated by the PTEN phosphatase (108,109,111). In TNBC, this pathway is active in 9.6% of patients due to the loss of PTEN activity (110) (Fig. 4B). Therefore, studies using PI3K inhibitors have been conducted in patients with TNBC (Table II), such as the LOTUS trial (NCT02162719), which is a phase-II trial evaluating ipatasertib in 124 patients (ORR in the PTEN^low^ group was 48% compared with patients with PTEN expression) (112). The oral pan-PI3K inhibitor buparlisib has also been used in combination with paclitaxel in a phase-II trial (NCT01572727) involving metastatic Her2-negative patients; the ORR was 22.6% compared with placebo and paclitaxel (113).

Capivasertib and AZD5363 are AKT inhibitors that are currently being investigated for PFS in patients with metastatic TNBC in the CAPItello-290 (NCT03997123) and PAKT (NCT02423603) trials, respectively (114,115). In the Phase-II trial under neoadjuvant setting, mTOR inhibitor and chemotherapy combined did not show any effect in early TNBC treatment (116). The mTOR inhibitors temsirolimus or everolimus in combination with doxorubicin and bevacizumab displayed an objective response rate of 21% in mesenchymal subtype of TNBC (117).

#### AR inhibitors for AR-overexpressing TNBC

AR belongs to the nuclear steroid hormone family of receptors, is highly expressed in the LAR subtype of TNBC (118). AR antagonists have shown an effect *in vitro* and *in vivo* in the LAR type (Fig. 4C). Gucalp *et al* (119) used the AR inhibitor bicalutamide in a phase-II trial involving 424 AR-positive patients, which showed a clinical benefit rate of 19% and a median progression-free survival of 12 weeks (119). Among the ongoing clinical trials, Bicalutamide treatment response is being compared to standard chemotherapy in patients with metastatic TNBC in an ongoing phase-III as the first line of therapy (NCT03055312). Enzalutamide is another AR antagonist with which a phase-II trial (NCT01889238) was conducted in AR-positive patients with advanced TNBC, in which a clinical benefit of 25% was observed (120). Androgen-driven gene expression signature (Dx-signature) stratified patients into a Dx-positive and a Dx-negative group. Dx-positive patients had an improved response to enzalutamide compared with Dx-negative patients (120,121). In AR-positive patients with early-stage TNBC, enzalutamide is currently under investigation both as a monotherapy (NCT02750358) and in combination with paclitaxel (NCT02689427). Around 40% of AR-positive TNBC patients show activation of the PI3K-AKT pathway (122). Therefore, the combined effect of enzalutamide and the PI3K inhibitor taselisib was evaluated in the TBRC032 trial(NCT02457910) where CBR was 35.7% (123). Further details are provided in Table II.

#### Antibody-drug conjugates targeting surface antigens

Antibody-drug conjugates (ADC) are made up of a linker, an inhibitor, and an antibody. The antibody is selected to be specific to cell surface molecules of cancer cells and not normal cells. The payload of cytotoxic agents must be potent to kill the cancer cell. Usually, a stable molecule is used as a linker that will bind strongly to the inhibitor (124,125) (Fig. 4D). Elevated expression of tumor-associated calcium-linked signal-transducer two cell surface glycoprotein (Trop-2) has been reported in TNBC and often correlated with poor prognosis (126). Sacituzumab Govitecan (IMMU-132) is an ADC used to target Trop-2 that delivers a topoisomerase-I inhibiting payload resulting in DSBs. Bardia *et al* (127) conducted a phase-I/II study involving patients with advanced-stage TNBC who had previously received two lines of treatment, and the ORR was 33.33%. A phase-III study (NCT02574455) of sacituzumab govitecan in relapsed patients with TNBC is ongoing. SKB264 is another anti-Trop2 currently under investigation in the NCT04152499 phase-I trial with metastatic TNBC patients (128). Another ADC, ladiratuzumab vedotin, an immunoglobulin G1 antibody with a microtubule inhibitor (MMAE), has shown an ORR of 25% of patients with TNBC (129).

#### Inhibitors targeting other signaling pathways

In addition to PARP and PI3K inhibitors, inhibitors of other molecular targets are being investigated in TNBC. HDAC inhibitors are currently being investigated as monotherapy (NCT02623751) and in combination with cisplatin (NCT02393794). Various Ataxia Telangiectasia and Rad3-Related Protein (ATR) and Wee inhibitors are also in clinical trials for TNBC (1). MEK inhibitors and inhibitors of cell cycle-regulating agents, such as Aurora kinase, showed antitumor effects in animal xenografts (130,131). Palbociclib, a cyclin-dependent kinase 4/6 inhibitor, was used in a phase-I study along with paclitaxel in patients with metastatic TNBC (n=9). Clinical benefit was experienced in one-third of the patients (132). BCL2 inhibitors in TNBC cell lines have shown to decrease cell proliferation (133). In TNBC cells, BCL2 expression is high (134). Therefore, BCL2 inhibitors should be further investigated for their impact as monotherapy and in combination.

#### Immunotherapy as monotherapy and combination therapy for TNBC

In the last decade, substantial evidence has been generated describing the immune system's role in guiding the disease progression of TNBC (135). It is one of the rapidly progressing areas of breast cancer research. The T cell receptor (TCR) recognizes antigen presented on major histocompatibility complex molecules by cancer cells (136). It is followed by signaling from co-stimulatory factors such as CD28, modulated by immune-checkpoint (co-inhibitory) molecules (137). In TNBC, programmed death-ligand 1 (PD-L1) functions as a critical mediator of the balance and escape stages of cancer immunoediting (138–140). Around 20% of TNBC tumors express PD-L1, which is associated with poor prognostic features, such as higher grade, HER2-positive status, ER-negative status and large tumor size (141). Quantification of PD-L1 can be carried out on immune cells or tumor cells using immunohistochemistry (141–143). Studies have suggested that PD-L1 expression varies depending on the stage of TNBC and cell type (141–143). Expression of PD-L1 in TNBC has been associated with improved pCR (50% vs. 21%) (39,144). Along with PD-L1, TILs are also high in number in TNBC (144,145). TILs are considered to be a good prognostic marker in TNBC (146). Inhibitors of PD-1/PD-L1 block the interaction between PD-1 and PD-L1, thereby initiating a positive immune response that results in tumor killing (123). Over the last few years, immune checkpoint inhibitors (CPIs) have been in the limelight due to improved efficacy shown during clinical trials (Fig. 4E). Pembrolizumab (NCT04191135 and NCT01042379), nivolumab (NCT03818685 and NCT03414684), atezolizumab (NCT03281954 and NCT03498716) and durvalumab (NCT03167619 and NCT03616886) are some of the CPIs currently used in ongoing clinical trials for TNBC (147). The IMpassion130 trial (NCT02425891) evaluated the use of atezolizumab with paclitaxel as the first line of therapy for patients with metastatic TNBC (n=901), showing PD-L1 positivity. Atezolizumab is a PD-LA inhibitor that blocks the interaction between PD-L1 and PD-1, thereby promoting T cell activity. It is now an FDA-approved drug for PD-L1-positive patients with TNBC (148). The KEYNOTE-119 phase-III clinical trial (149) evaluated pembrolizumab's effect as monotherapy in patients with metastatic TNBC vs. physician's choice chemotherapy (capecitabine, vinorelbine, gemcitabine, or eribulin). The OS of this study was not encouraging (149). In the recent trial KEYNOTE-355 (NCT02819518), PD-L1-positive patients with metastatic TNBC showed improved PFS when pembrolizumab was given in combination with chemotherapy, in comparison with patients given chemotherapy alone (150). Currently, two trials, IMpassion131 (NCT03125902) and IMpassion132 (NCT03371017), are being carried out: The former is investigating the outcomes for paclitaxel and atezolizumab in untreated metastatic patients who are PD-L1 positive, while the latter is for atezolizumab along with chemotherapy (gemcitabine, capecitabine and carboplatin) in early relapsing recurrent patients with TNBC (PD-L1 positive). For early-stage breast cancer, the KEYNOTE-173 phase-Ib trial evaluated pembrolizumab along with taxane and anthracycline neoadjuvant therapy, which resulted in an ORR of 100% (151). The ISPY-2 trial was a phase-III trial evaluating pembrolizumab in combination with chemotherapy (vs. placebo) in patients with stage-II/III TNBC, which demonstrated an ORR of 60 and 20%, respectively (152). The SWOG S1418 (NCT02954874) trial is investigating anti-PD-1/-PD-L1 in the adjuvant setting for a year in order to determine whether there is an improvement in DFS. The NSABP B-59 (NCT03281954) and IMpassion030 (NCT03498716) trials are addressing whether the combination of neoadjuvant/adjuvant chemotherapy and atezolizumab might improve DFS compared with chemotherapy alone (153).

## 8. Non-coding RNA as therapy

Sequencing of all the RNA species in a given cell using RNA-seq identified several RNA species, including mRNA. The two major classes of non-coding RNA studied in TNBC development and treatment are miRNA and Long non-coding RNA.

MicroRNA (miRNA/miR) is a small non-coding RNA, usually 20–22 nucleotides in length, regulating gene expression. miRNA is known to bind to the 3′untranslated region of mRNA. This binding either degrades mRNA or represses translation (154). miRNA is a key player in tumorigenesis, stemness, and drug resistance in TNBC (155–157). For instance, tumour suppressor miRNAs, involved in tumour development, miR-190a, miR-136-5p, miR-126-5p, miR-135b-5p and miR-182-5p are downregulated in TNBC (158). miR-22 is downregulation in TNBC, is associated with migration and metastasis. miR-22 exerts its effect through eukaryotic elongation factor 2 kinase (eEF2K) expression, which activates PI3K signaling pathway (159). Also, oncosuppressor, miR-200b, activate target genes like SRY-box transcription factor 2 (SOX2), CD133, and zinc finger E-box binding homeobox 1 (ZEB1), aiding in migration and invasion and stemness (157,160). High expression of miR-95 in TNBC indicates radiotherapy resistance that occurs by targeting sphingosine-1-phosphate signaling (161). Downregulated miR-449 upregulates CDK2, CCNE2 causing doxorubicin resistancein TNBC (162,163) (Table III). Multiple studies also show that miRNAs are expressed in different stages of TNBC Multiple studies also show that miRNAs are expressed in different stages of TNBC (164–166). These studies give hope for miRNA-based therapies, as the use of miRNA mimics or inhibitor oligonucleotides could serve as a therapeutic approach for TNBC (167). A study conducted by Shu *et al* (168) used miR-21 combined with aptamer targeting EGFR, blocking tumor growth in murine models. Yin *et al* (169) designed an RNA aptamer bound to CD133 with a sequence complementary to miR-21 carried by a three-way junction motif scaffold that reduced cell migration in TNBC cells (169). Non-coding RNA is being pursued as one of the TNBC therapy.

lncRNA (long non-coding RNA), ~200 nucleotides in length, regulates gene expression at the epigenetic, transcription, post-transcription levels, and post-translation modification (16). The long intergenic non-coding RNA for kinase activation activates HIF-1α by phosphorylating it via leucine-rich repeat kinase 2to promote glycolysis and tumorigenesis in TNBC (170). Yang *et al* (171) demonstrated the involvement of POU domain class 3 transcription factor 3 (POU3F3) in inhibiting apoptosis and promoting proliferation in TNBC (171). Nuclear paraspeckle assembly transcript 1 (NEAT1) plays a role in TNBC metastasis (172–174). Some lncRNAs (HOTAIR, LncRNA-ATB, LincRNA-ROR) are known to be co-expressed with transcription factors involved in EMT and proliferation (175). Vaidya *et al* (176) demonstrated that nanoparticle-mediated transfer of RNA interference molecules targeting differentiation antagonizing non-protein coding RNA, a lncRNA that is enriched in TNBC, showed some efficacy in a murine xenograft model of TNBC (Table III). These studies have shed light on the use of antisense oligonucleotides against oncogenic lncRNA as a potential approach to TNBC therapy.

## 9. Conclusions

TNBC is associated with poor prognosis compared to other breast cancer subtypes, and its treatment remains challenging. New technology and tools have provided insight into the molecular mechanism of the disease. This knowledge has led to the identification of druggable targets and the development of biomarker-driven therapy. The FDA-approved drugs for TNBC to date include PARP inhibitors for patients with BRCA1/2 mutations and atezolizumab for PD-L1^+^ tumors. Emerging targeted therapies have given hope for the treatment of TNBC. The inclusion of immunotherapy has shown promising results. Additionally, attempts to identify combinations that work effectively against TNBC are ongoing. A combination of the molecular profiles, including non-coding RNA and histology, has improved the prognosis and guided the treatment for TNBC.

## Figures and Tables

**Table I.** Molecular classification of triple-negative breast cancer.

First author, year	Country or region	Method	Classification	Features/Pathways enriched	(Refs.)
Perue *et al*, 2000	Norway and Stanford	Microarray ScanArray 3000; PAM50	Luminal A	Estrogen receptor and transcription factors (GATA, X-box binding proteins, EST downregulation)	(3)
			Luminal B	Estrogen receptor and transcription factors (GATA, X-box binding proteins)	
			Her2^+^	ERBB2 and GRB7 overexpression	
			Normal-like	Overexpression of adipose tissue and other non-epithelial cell types expressed genes	
			Basal-like	Keratin 5 and −17, laminin and fatty acid binding protein 7 overexpression	
Lehmann *et al*, 2014	Sweden, UK, Netherlands, USA, Singapore/Belgium	Affymetrix	Basal-like 1	Cell cycle, DNA replication reactome, RNA polymerase, and	(42)
			Luminal Basal-like 2	EGF pathway, NGF pathway, MET pathway, Wnt/β-catenin, and IGF1R pathway	
			Mesenchymal	Cell motility, ECM receptor interaction and cell differentiation pathways	
			Mesenchymal stem cell-like	Cell motility, cellular differentiation, growth pathway, inositol phosphate metabolism, EGFR, PDGF, calcium signalling,	
			Immunomodulatory subtype	Immune cell signalling cytokine signalling, antigen processing and presentation, and signalling through core immune signal transduction pathways	
			Androgen receptor subtype	Steroid synthesis, porphyrin metabolism, and androgen/oestrogen metabolism	
Curtis *et al*, 2012	UK and Canada	Affymetrix; Illumina	Integrative cluster 1	17q23/20q cis-acting	(47)
	(METABRIC)	HT-12 v3 platform	Integrative cluster 2	11q13/14 cis-acting	
			Integrative cluster 3	Low genomic instability	
			Integrative cluster 4	CNA-devoid	
			Integrative cluster 5	ERBB2-amplified	
			Integrative cluster 6	8p12 cis-acting	
			Integrative cluster 7	16p gain/16q loss, 8q amplification	
			Integrative cluster 8	1q gain/16q loss	
			Integrative cluster 9	8q cis-acting/20q-amplified	
			Integrative cluster 10	cis-acting alterations 5 loss/8q gain/10p gain/12p gain	
Burstein *et al*, 2015	USA and European	Affymetrix	Luminal-AR	androgen receptor, oestrogen receptor, prolactin, and ERBB4 signalling	(43)
			Mesenchymal	cell cycle, mismatch repair and DNA damage networks, and hereditary breast cancer signalling pathways	
			Basal-like immune-suppressed	downregulation of B cell, T cell and natural killer cell immune-regulating pathways and cytokine pathways	
			Basal-like immune-activated	upregulation of genes controlling B cell, T cell, and natural killer cell function	
Liu *et al*, 2016	China	Affymetrix	Immunomodulatory subtype	Cytokine-cytokine receptor interaction, T cell receptor signalling pathway, B cell receptor signalling pathway, chemokine signalling pathway	(45)
			Luminal-AR	Steroid hormone biosynthesis, Porphyrin and chlorophyll metabolism, PPAR signalling pathway, Androgen and oestrogen metabolism	
			Mesenchymal	ECM-receptor interaction, Focal adhesion, TGF-β signalling pathway, ABC transporter, Adipocytokine signalling pathway	
			Basal-like immune-suppressed	Mitotic cell cycle, Mitotic prometaphase, M phase of mitotic cell cycle, DNA replication, DNA repair	

ABC, ATP-binding cassette; GRB2, growth factor receptor bound protein 2; SNP, single nucleotide polymorphism; ECM, extracellular matrix; CAN, copy number alteration; PPAR, peroxisome proliferator-activated receptor; EST, expressed sequence tag.

**Table II.** Summary of clinical trials of different group of inhibitors used as targeted therapy in TNBC.

A, PARP inhibitors					

Drug	Trial	Phase	Stage	Disease setting	Results
carboplatin + paclitaxel + veliparib → AC vs. carboplatin + paclitaxel + placebo → AC vs. placebo + placebo + placebo → AC	BrighTNess, NCT02032277	III	Early	Stage II/III TNBC	ORR 58% vs. 53% vs. 31%
standard NACT + olaparib vs. standard	GeparOla, NCT02789332 NACT + carboplatin AUC2	II	Early	Stage I–III HER2^−^ BC with gBRCA1/2m and/or HRD	pCR 55.1% vs. 48.6%
talazoparib	NCT02282345	II	Early	Stage I–III gBRCA1/2m BC	Ongoing
veliparib + carboplatin → standard NACT vs. standard NACT	I-SPY, NCT01042379	II	Early	Stage II–III TNBC	Ongoing
olaparib + carboplatin + paclitaxel → AC/EC vs. Paclitaxel + carboplatin → AC/EC	PARTNER, NCT03150576	II/III	Early	Stage II/III TNBC and/or gBRCAm BC	Ongoing
olaparib vs. placebo	OlympiA, NCT02032823	III	Early	HER2^−^ BC gBRCA1/2m	Ongoing
olaparib + durvalumab + AZD6738	PHOENIX, NCT03740893	II	Early	Stage II/III TNBC	Ongoing
olaparib vs. PCT	OlympiAD NCT02000622	III	Advanced	Metastatic TNBC (gBRCA1/2^+^), prior lines	ORR 59.9% vs. 29.9%
talazoparib vs. PCT	EMBRACA, NCT01945775	III	Advanced	Metastatic TNBC (gBRCA1/2^+^), prior lines	ORR 62.6% vs. 27.2%
niraparib vs. PCT	BRAVO, NCT01905592	III	Advanced	Metastatic TNBC (gBRCA1/2^+^), prior lines	Ongoing
veliparib + paclitaxel + carboplatin vs. placebo + Paclitaxel + carboplatin	BROCADE3, NCT02163694	III	Advanced	Metastatic TNBC (gBRCA1/2^+^), prior lines	Ongoing
niraparib + pembrolizumab	TOPACIO NCT02657889	I/II	Advanced	Metastatic TNBC	Ongoing
olaparib vs. olaparib + ceralasertib vs. olaparib + adavosertib	NCT03330847	II	Advanced	Metastatic TNBC	Ongoing
olaparib + durvalumab + bevacizumab	MEDIOLA, NCT02734004	I/II	Advanced	gBRCAm metastatic HER-2^−^ BC	Ongoing
olaparib + durvalumab	NCT03801369	II	Advanced	Metastatic TNBC	Ongoing
talazoparib + avelumab	NCT03330405	II	Advanced	Metastatic TNBC	Ongoing
olaparib + durvalumab	NCT03167619	II	Advanced	Metastatic TNBC	Ongoing
olaparib	DORA, NCT00679783	II	Advanced	Metastatic TNBC	Ongoing
talazoparib + ZEN003694	NCT03901469	II	Advanced	Metastatic TNBC	Ongoing
talazoparib	NCT02401347	II	Advanced	BRCA1/2 wild-type HER2^−^ BC	Ongoing
veliparib + cisplatin	NCT02595905	II	Advanced	Metastatic TNBC	Ongoing
pembrolizumab + olaparib + gemcitabine + carboplatin	NCT04191135	II/III	Advanced	Metastatic TNBC	Ongoing

**B, PI3K inhibitors**					

**Drug**	**Trial**	**Phase**	**Stage**	**Disease setting**	**Results**

iptasertib + paclitaxel vs. placebo + paclitaxel	LOTUS, NCT02162719	II	Advanced	Advanced TNBC	ORR 40% vs. 32%
buparlisib +paclitaxel vs. placebo + paclitaxel	BELLE-4, NCT01572727	II	Metastatic	Metastatic Her-2^−^	ORR 22.6% vs. 27%
capivasertib + paclitaxel vs. placebo + paclitaxel	PAKT, NCT02423603	II	Metastatic	Metastatic TNBC	Ongoing
Tak-228 + Tak-117 + cisplatin + Nab-paclitaxel	NCT03193853	II	Metastatic	Metastatic TNBC	Ongoing
LY3023414 + prexasertib	ExIST, NCT04032080	II	Metastatic	Metastatic TNBC	Ongoing
everolimus + carboplatin	NCT02531932	II	Metastatic	Metastatic TNBC	Ongoing
ipatasertib + paclitaxel	IPATunity130; NCT03337724	II/III	Metastatic	Metastatic TNBC	Ongoing
alpelisib + Nab-paclitaxel	NCT04216472	II	Metastatic	Metastatic TNBC	Ongoing
capivasertib + paclitaxel	CapItello290, NCT03997123	III	Metastatic	Metastatic TNBC	Ongoing
IPI-549 + atezolizumab + bevacizumab + Nab-paclitaxel	MARIO-3, NCT03961698	II	Metastatic	Metastatic TNBC	Ongoing
gedatolisib + talazoparib	NCT03911973	II	Metastatic	Metastatic TNBC	Ongoing
vistusertib + selumetinib	TORCMEK NCT02583542	II	Metastatic	Metastatic TNBC	Ongoing
capivasertib +ceralasertib + adavosertib + olaparib	OLAPCO NCT02576444	II	Metastatic	Metastatic TNBC	Ongoing

**C, AR Antagonists**					

**Drug**	**Trial**	**Phase**	**Stage**	**Disease setting**	**Results**

enzalutamide	NCT02750358	II	Early	TNBC	Ongoing
enzalutamide + paclitaxel	NCT02689427	II	Early	Stage I–III	Ongoing
bicalutamide	NCT03055312	II	Advanced	Metastatic TNBC	CBR 19%
enzalutamide	TBCRC011, NCT01889238	II	Advanced	Metastatic Her-2^−^	CBR 25%
bicalutamide	NCT00468715	II	Advanced	Metastatic BC	Ongoing
abiraterone acetate + prednisone	NCT01842321	II	Advanced	Metastatic Her-2^−^	Ongoing
palbociclib + bicalutamide	NCT02605486	I/II	Advanced	Metastatic BC	Ongoing
orteronel	NCT01990209	II	Advanced	Metastatic TNBC	Ongoing
enobosarm + pembrolizumab	NCT02971761	II	Advanced	AR^+^ Metastatic TNBC	Ongoing
bicalutamide + palbociclib	NCT02605486	II	Advanced	AR^+^ Metastatic BC	Ongoing
enzalutamide + taselisib	NCT02457910	I/II	Advanced	AR^+^ Metastatic TNBC	Ongoing
enzalutamide + alpelisib	NCT03207529	II	Advanced	AR^+^ PTEN^+^ Metastatic BC	Ongoing
bicalutamide	SYSUCC-007, NCT03055312	III	Advanced	AR^+^ Metastatic TNBC	Ongoing
enzalutamide	NCT02750358	II	Advanced	AR^+^ Metastatic TNBC	Ongoing
bicalutamide + ribociclib	NCT03090165	I/II	Advanced	AR^+^ Metastatic TNBC	Ongoing
darolutamide + capecitabine	START, NCT03383679	II	Advanced	Metastatic BC	Ongoing
orteronel	NCT01990209	II	Advanced	Metastatic BC	Ongoing

**D, ADCs**					

**Drug**	**Trial**	**Phase**	**Stage**	**Disease setting**	**Results**

sacituzumab govitecan-hziy (topoisimerase-1 inhibitor SN-38), Trop2 ADC	NCT01631552	I/II	Advanced	Advanced TNBC	ORR 33%
ladiratuzumab vedotin, MMAE microtubule inhibitor, LIV-1	NCT01969643	I	Advanced	Advanced TNBC	ORR 25%
sacituzumab govitecan chemotherapy	ASCENT NCT02574455	II	Advanced	Advanced TNBC	Ongoing
CAB-ROR2-ADC+BA3021	NCT03504488	I/II	Metastatic	Metastatic TNBC	Ongoing
SKB264	A264, NCT04152499	I/II	Metastatic	Metastatic TNBC	Ongoing
enfortumab vedotin	EV-202, NCT04225117	II	Metastatic	Metastatic TNBC	Ongoing

**E, Immune Checkpoint Inhibitors**					

**Drug**	**Trial**	**Phase**	**Stage**	**Disease setting**	**Results**

durvalumab + Nab-paclitaxel → EC vs. placebo + Nab-paclitaxel → EC	GeparNuevo, NCT02685059	II	Early	Stage II	pCR 53.4% vs. 44.2%
Nab-paclitaxel + carboplatin + pembrolizumab → AC+ pembrolizumab vs. placebo + Nab-paclitaxel + carboplatin → AC	KEYNOTE-173, NCT02622074	I	Early	T2/T3 88.3%, ≥N1 66.7%	pCR 60%
pembrolizumab + chemotherapy vs. placebo + chemotherapy	KEYNOTE-522, NCT0303648	III	Early	T1cN1-2 or T2-4N0-N2	pCR 64.8% vs. 51.2%
carboplatin + nab-paclitaxel + atezolizumab → surgery → AC/EC/FEC	NeoTRIPaPDL1, NCT02620280	III	Early	T1cN1, T2N1, T3N0	pCR 43.5% vs. 40.8%
pembrolizumab + paclitaxel → AC vs. placebo + Paclitaxel → AC	ISPY-2, NCT01042379	II	Early	Stage II/III	Ongoing
pembrolizumab	SWOG1418/BR006, NCT02954874	III	Early	ypT ≥1 cm or ypN1-3, TNBC	Ongoing
avelumab	A-BRAVE, NCT02926196	III	Early	ypT>1 mm or ypN1-3 or IIB-III	Ongoing
atezolizumab + paclitaxel + carboplatin → atezolizumab + AC/EC vs. paclitaxel + carboplatin → AC/EC	NSABP B 59, NCT03281954	III	Early	≥ T2N0 or ≥ T1cN1	Ongoing
aezolizumab + paclitaxel → atezolizumab + AC/EC vs. paclitaxel → AC/EC	IMpassion030, NCT03498716	III	Early	II–III	Ongoing
atezolizumab + Nab paclitaxel → atezolizumab + AC vs. placebo+ Nab paclitaxel → placebo + AC	IMpassion031, NCT03197935	III	Early	cT2-cT4, cN0-cN3, cM0	Ongoing
pembrolizumab vs. PTC	KEYNOTE-119, NCT02555657	III	Advanced	Metastatic TNBC	Negative
atezolizumab + Nab paclitaxel vs. placebo + Nab paclitaxel	IMpassion130, NCT02425891	III	Advanced	Metastatic TNBC	OS 7.2 vs. 5.5 months
pembrolizumab	KEYNOTE-012, NCT01848834	I	Advanced	Metastatic TNBC	ORR 18.5%
pembrolizumab	KEYNOTE-086, NCT02447003	II	Advanced	Metastatic TNBC	ORR ~5%
avelumab	JAVELIN, NCT01772004	I	Advanced	Metastatic TNBC	ORR 21.6%
atezolizumab	NCT01375842	I	Advanced	Metastatic TNBC	ORR 10%
atezolizumab + paclitaxel vs. placebo + paclitaxel	IMpassion131, NCT03125902	III	Metastatic	Metastatic TNBC	Ongoing
atezolizumab + gemcitabine + capecitabine + carboplatin vs. placebo + gemcitabine + capecitabine + carboplatin	IMpassion132, NCT03371017	III	Metastatic	Metastatic TNBC	Ongoing
pembrolizumab + Nab-paclitaxel + paclitaxel + gemcitabine + carboplatin vs. placebo + Nab-paclitaxel +paclitaxel + gemcitabine + carboplatin	KEYNOTE-355, NCT02819518	III	Metastatic	Metastatic TNBC	Ongoing
pembrolizumab + eribulin	ENHANCE-1, NCT02513472	I/II	Metastatic	Metastatic TNBC	Ongoing
NKTR-214 1 nivolumab	PIVOT-02 NCT02983045	II	Metastatic	Metastatic TNBC	Ongoing
Intratumoral c-MET mRNA CAR T cells	NCT01837602	I	Metastatic	Metastatic TNBC	Ongoing

**F, Conventional platinum agents**					

**Drug**	**Trial**	**Phase**	**Stage**	**Disease setting**	**Results**

carboplatin + bevacizumab + standard NAC vs. bevacizumab + standard NAC	GeparSixto, NCT01426880	II	Early	Stage II/III/IV	pCR 53.2% vs. 36.9%
cisplatin + paclitaxel + everolimus vs. cisplatin + paclitaxel + placebo	NCT00930930	II	Early	Stage II/III, TNBC	pCR 36% vs. 48%
paclitaxel + carboplatin vs. paclitaxel + epirubicin	NCT01276769	II	Early	Stage II/III, TNBC	pCR 38.6% vs. 14.0%
cisplatin + paclitaxel	SHPD001, NCT02199418	II	Early	TNBC	pCR 64.7%
gemcitabine + carboplatin + iniparib	PreECOG 0105NCT00813956	II	Early	Stage I–IIIA	pCR 62.4% vs. 22.3%
paclitaxel + carboplatin + bevacizumab → dose-dense AC vs. standard NAC	CALGB40603, NCT00861705	II	Advanced	Locally advanced TNBC	pCR 62.4% vs. 22.3%

ORR, overall response rate; CBR, clinical benefit rate; OS, overall survival; PCT, physician's choice therapy; PFS, progression-free survival; AC, doxorubicin/cyclophosphamide; ADC, antibody-drug conjugate; EC, epirubicin/cyclophosphamide; pCR, pathological complete response; FEC, fluorouracil/epirubicin/cyclophosphamide; AR, androgen receptor; Her2, human epidermal growth factor receptor 2; gBRCA1/2, germline BRCA1/2; gBRCA1/m, gBRCA1/2-mutated; TNBC, triple-negative breast cancer; NAC, doxorubicin and cyclophosphamide; NACT, neoadjuvant chemotherapy; AUC2, area under the free carboplatin plasma concentration vs. time curve, value 2; HRD, homologous recombination deficiency; cT, clinical classified tumour; cN, clinical node staging; yPT, pathologic post-therapy tumour stage; yPN, pathologic post-therapy node stage; CM, clinical modification.

**Table III.** Role of miRNA and lncRNA expressed in triple negative breast cancer.

A, miRNA			

First author, year	Names	Role in TNBC	(Refs.)
Gorur *et al*, 2021; Pang *et al*, 2018;	miR-22 and miR-200 family	Epithelial-to-mesenchymal transition	(159,160)
Lyng *et al*, 2012	miR-190a, miR-136-5p, miR-126-5p, miR-135b-5p, miR-182-5p	Tumorigenesis	(158)
Huang *et al*, 2013; Tormo *et al*, 2019	miR-95, miR-449, and miR15a/16	Drug resistance	(161,162)

**B, lncRNA**			

**First author, year**	**Names**	**Role in TNBC**	**(Refs.)**

Lin *et al*, 2016	LINKA	Glycolysis and tumorigenesis	(170)
Jiang *et al*, 2018; Ke *et al*, 2016	NEAT1	Migration, invasion and apoptosis	(172,173)
Yang *et al*, 2019	POU3F3	Inhibits apoptosis	(171)
Sha *et al*, 2017	DANCR	Inhibits apoptosis	(177)

miR/miRNA, microRNA; TNBC, triple-negative breast cancer; LINKA, long intergenic non-coding RNA for kinase activation; NEAT1, nuclear paraspeckle assembly transcript 1; POU3F3, POU domain class 3 transcription factor 3; differentiation antagonizing non-protein coding RNA; DANCR, differentiation antagonizing nonprotein coding RNA.

## Data Availability

Not applicable.

## Abbreviations

TNBC: triple-negative breast cancer
FGFR: fibroblast growth factor receptor
ORR: overall response rate
OS: overall survival
PFS: progression free survival
PCT: physician's choice therapy
